# Advancing Pediatric Oncology Rehabilitation: Survey Findings of Health Professionals’ Perceptions of Barriers to Care and a Framework for Action

**DOI:** 10.3390/cancers15030693

**Published:** 2023-01-23

**Authors:** Paula A. Ospina, Lesley Pritchard, David D. Eisenstat, Margaret L. McNeely

**Affiliations:** 1 Department of Physical Therapy, University of Alberta, Edmonton, AB T6G 2G4, Canada; 2 Children’s Cancer Centre, Royal Children’s Hospital, Parkville, VIC 3052, Australia; 3 Murdoch Children’s Research Institute, Parkville, VIC 3052, Australia; 4 Department of Paediatrics, University of Melbourne, Parkville, VIC 3052, Australia; 5 Department of Oncology, Faculty of Medicine, University of Alberta, Edmonton, AB T6G 2R3, Canada; 6 Cancer Care Alberta, Alberta Health Services, Edmonton, AB T5J 3E4, Canada

**Keywords:** cancer, pediatrics, adolescent, rehabilitation, physical therapy, referrals, framework, barriers, healthcare provider

## Abstract

**Simple Summary:**

Children with cancer are at high risk of developing physical impairments, which can be minimized through participation in rehabilitation programs. Unfortunately, the literature suggests that pediatric oncology rehabilitation services are underutilized and that few children are referred to the service. This study aimed to explore referral practices to pediatric rehabilitation in Canada, identify healthcare providers’ perspectives of barriers to service provision, and propose a framework for action to support the advancement of rehabilitation programs for children with cancer. Canadian healthcare providers working in pediatric oncology participated in a web-based survey. Results demonstrated that medical referrals to rehabilitation services occur mostly when the child presents with, or is at risk of, severe deficits due to surgery. Healthcare providers identified a large number of barriers to rehabilitation. A framework for action is proposed, comprising key strategies to enhance rehabilitation services for children with cancer and to inform future research.

**Abstract:**

Purpose: To explore pediatric oncology referral practices, gather healthcare providers’ perspectives of barriers to access and provision of rehabilitation service across Canada, and inform a framework for action to optimize rehabilitation care and inform future research. Methods: A cross-sectional survey was conducted with Canadian healthcare professionals (HCPs) working in pediatric oncology. Results: A total of 54 responses were received, and 34 corresponded to HCPs who refer children with cancer to rehabilitation services. Results suggest that approximately 25% of children are referred to rehabilitation services, primarily when the child presents with, or is at risk of, significant functional disability due to surgery. A primary barrier to service provision identified across HCPs included a lack of funding and resources. Medical professionals further identified a lack of specialized pediatric oncology rehabilitation services, whereas rehabilitation professionals identified the lack of pediatric oncology specific space and equipment. Identified themes from open-ended survey questions include the need for (1) dedicated funding and resources, (2) improved access, and (3) the need for specialized pediatric oncology rehabilitation services. Conclusion: Several barriers exist in the Canadian healthcare context that impact the delivery of rehabilitation services for children with cancer. We propose a framework for action to advance clinical care and guide future research.

## 1. Introduction

Childhood cancer survival has increased, with a current 5-year survival rate of nearly 85% [1]. It is estimated that two thirds of children with cancer will develop at least one chronic or long-term adverse effect of cancer treatment [2], such as cancer-related fatigue [3], muscle weakness [4], peripheral neuropathy [5], deficits in balance and ambulation [6,7], and decreased joint range of motion [6]. During the active cancer treatment period, children may spend prolonged periods of time on bedrest [8], resulting in impairments in physical function that can lead to rapid deconditioning [9,10] and negatively impacting muscle mass and strength by 1.0% to 1.5% per day, or 10 to 15% per week [8]. The combined adverse effects to musculoskeletal, neurological, and cardiorespiratory systems, although potentially reversible, may persist or worsen over time [11]. Thus, as childhood cancer survival rates increase, there is also a growing need for rehabilitation services to attenuate the impact on the long-term physical function and quality of life of children with cancer [5,8,12].

### 1.1. Current State of Knowledge in Pediatric Oncology Rehabilitation

There is some evidence of the potential benefits of physical therapy (PT) for minimizing the severity of adverse effects and improving physical function of children and adolescents with cancer [13,14,15]. In 2019, we published a scoping review that explored the state of the research evidence on the benefits of PT for children and adolescents with cancer and identified priorities for future research [16]. A total of 12 articles were included in the review, comprising one randomized controlled trial, one pilot randomized trial, five pilot or feasibility studies, two prospective studies, one case series, one case report, and one retrospective study. Results revealed that, while the current research evidence is not at a level to inform effectiveness, hospital and home-based PT programs are feasible (i.e., completion rates) and show promise for children during and after cancer treatment.

Recent systematic reviews have evaluated the effects of exercise on physical function outcomes. Coombs et al. [13], published a systematic review that evaluated the effects of exercise and motor interventions on physical activity and motor outcomes in children diagnosed with acute lymphoblastic leukemia. A total of 19 studies were included, and the results support the benefit of exercise for fatigue, range of motion, physical activity levels, aerobic capacity, functional mobility, strength, coordination, and life participation [13]. Findings from a systematic review and meta-analysis of randomized controlled trials published by Shi et al. [17], examined the adherence of childhood cancer survivors to supervised exercise training interventions as well as the impact on health outcomes. Nine studies were included, and results demonstrated a high adherence (87%) to exercise interventions and improvements in muscle strength, physical activity levels, body mass index, and fatigue [17].

While research evidence supporting physical therapy and general physical exercise interventions for children with cancer has increased over time, limited literature exists supporting the benefit of rehabilitation disciplines such as occupational therapy and speech-language pathology, and current research evidence suggests underutilization of occupational therapy services in this population [18]. More recent reviews have examined the effectiveness of play therapy in children with cancer and other life-threatening conditions, as well as the factors influencing their participation in play [19,20]. Results suggest that introducing play therapy in the hospital setting can reduce anxiety and fear [19]; however, children’s ability to participate in play is influenced not only by their health condition, but by the opportunities provided to play and the availability of appropriate space and equipment [20].

Engaging children and adolescents with cancer in early rehabilitation programs may help minimize the occurrence and severity of treatment-related effects [21,22,23], as well as help to maintain and restore their ability to perform daily life activities and consequently improve their quality of life [24]. Unfortunately, research has shown that pediatric oncology rehabilitation services are underutilized. In a recent retrospective cohort study of 5488 children with acute lymphoblastic leukemia from 330 hospitals, only 27.2% overall and 58.9% with identified neuromuscular conditions received rehabilitation within the first year of hospital admission [25]. Moreover, a cross-sectional study evaluating the use of rehabilitation services to address late effects on 5+ year survivors of childhood cancer reported that, of 9289 survivors, only 9.3% reported receiving PT services [26]. In a survey exploring the percentage of hospitalized children receiving PT for the treatment of chemotherapy-induced peripheral neuropathy, only one third of children received treatment [27]. To our knowledge, no published research to date has explored the current service provision and referral patterns specific to pediatric oncology rehabilitation in Canada.

### 1.2. Objectives

The purposes of this study were to (1) explore the frequency of and reasons for referrals to pediatric oncology rehabilitation services, (2) identify existing barriers to access including barriers to provision of pediatric oncology rehabilitation programs, and (3) propose a framework to support advanced care and future research in pediatric oncology rehabilitation.

## 2. Materials and Methods

We conducted a cross-sectional web-based survey in a secure Research Electronic Data Capture (REDCap) service at the University of Alberta in Edmonton, Canada [28]. REDCap provides (1) an intuitive interface for validated data entry, (2) audit trails for tracking data manipulation and export procedures, (3) automated export procedures for seamless data downloads to common statistical packages, and (4) procedures for importing data from external sources. The study was approved by the Health Research Ethics Board of Alberta: Cancer Committee. Electronic informed consent was obtained from all participants. Details of the study design have been published previously [29].

The survey was administered between July and October 2017. HCPs who had provided and/or referred children and adolescents with cancer to rehabilitation services across Canada were eligible for the study. The survey was sent out through professional networking and organizations. Twenty-eight additional organizations, institutions, and facilities that provide cancer care were identified online and contacted. Information about the study was delivered via email to healthcare providers and distributed in the newsletters and e-blasts of the organizations and associations. To increase the response rate, we sent electronic reminders every three weeks, and respondents were asked to forward the survey communication to other HCPs who were working with the pediatric oncology population.

Our sample size was estimated based on a previous survey in oncology service provision in adults with cancer in Canada conducted by Canestraro et al. [30], (*n* = 62). Given the low incidence of pediatric cancers in Canada (less than 0.1% of the total cancer cases) [2], we anticipated fewer HCPs work in this field. Thus, we aimed to collect data from a representative sample of at least 30 respondents.

### 2.1. Instrument and Data Analysis

The survey was available in English and French (Table S1), and questions were designed based on previous studies conducted in cancer rehabilitation [30,31]. The survey included 30 questions, subdivided into three sections. The first section of the survey gathered demographic data including professional designation, location of practice, type of service, and length of experience in the field, as well data on numbers of children with cancer seen or referred in the respective facility per year. The second section included questions related to rehabilitation service provision and practice patterns. The final section included questions on barriers that impact the implementation of rehabilitation programs, as well as existing pediatric oncology-specific guidelines and programs in their work settings.

For analysis purposes, this publication includes results related to the barriers to service provision and referral patterns gathered from sections I and III of the web-based survey, corresponding to information provided by survey respondents who referred children and adolescents with cancer to rehabilitation services. Results from section II, involving data from rehabilitation professionals on the nature and extent of rehabilitation programs, are published elsewhere [29].

#### Variables

- Section I—Demographic information and referral patterns
  ○ Professional designation: HCPs’ professional designations.
  ○ Location of practice: Canadian province where HCPs primarily work.
  ○ Type of service: Primary type of work setting where HCPs work at.
  ○ Length of experience: Total number of months or years of HCPs’ experience in pediatric oncology.
  ○ Number of children seen: Total number of children with cancer seen each year by HCPs.
  ○ Number of children referred to rehabilitation: Total number of children of cancer referred to rehabilitation services each year by HCPs.
  ○ Frequency of referral to rehabilitation: HCPs’ perceptions of the frequency of referral of children with cancer to rehabilitation services.
  ○ Percentage of children that received rehabilitation: Percentage of children who ended up receiving rehabilitation, following referral to the service.
  ○ Reasons children did not receive rehabilitation: Reasons why children did not receive rehabilitation, following referral to the service.
  ○ Referral location: Type of location where children with cancer were referred to rehabilitation services.
  ○ Reasons that prompt referral to rehabilitation: Reasons that prompt HCPs’ referral of children with cancer to rehabilitation services.
- Section III—Barriers to rehabilitation and availability of programs and guidelines
  ○ Availability of rehabilitation programs and clinical practice guidelines: Number of work settings with pediatric oncology rehabilitation programs and clinical practice guidelines in place.
  ○ Reasons for not having a program: Reasons why the work settings do not have a pediatric oncology rehabilitation program.
  ○ Barriers to rehabilitation programs: Existing barriers to pediatric oncology rehabilitation programs from the perspective of HCPs.

The data collected consisted of continuous and categorical responses as well as open-ended questions. Data were imported into Microsoft Excel for calculating percentages (%), frequencies (f), and proportions for data description. Two study investigators categorized open-ended questions into themes using framework analysis and coded responses to provide context for the findings and inform a framework for action [32]. Framework analysis provides a means to answer specific questions with actionable outcomes, lending itself well to informing strategies for clinical research and practice [32,33]. To analyze data, one study investigator quantified the frequency of responses and grouped responses into categories. A second investigator verified the calculations and categories. The two investigators then explored themes and patterns and interpreted findings in the context of the survey responses.

## 3. Results

**Figure 1:**
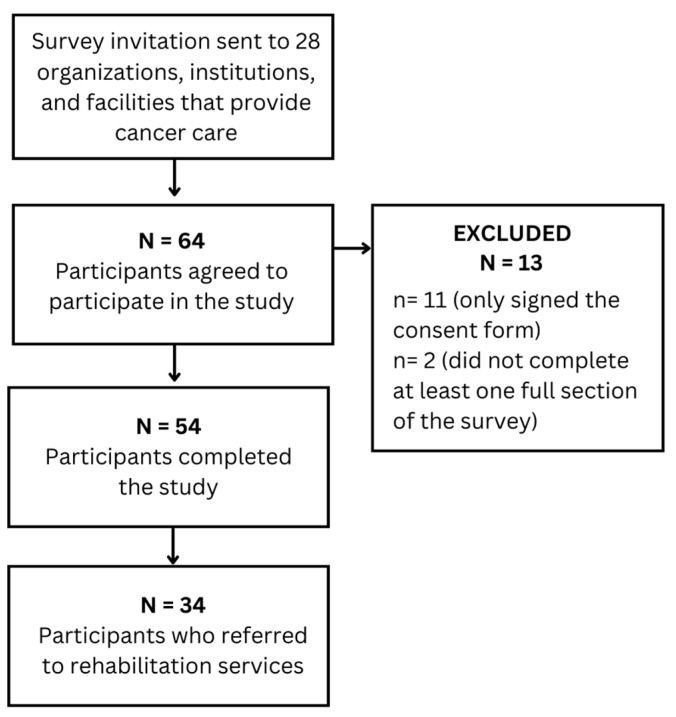
Flow diagram of survey respondents.

A total of 67 HPCs agreed to participate in the survey. Eleven participants signed the consent form and did not complete the survey, and two participants did not complete at least one full section of the survey. Of the 54 survey responses included in the study, 34 respondents were HCPs who refer children to pediatric oncology rehabilitation services (Figure 1).

The number of participants who provided responses in each section is indicated in the section headings and tables. Results are divided into Group 1—medical team (oncologists, oncology residents, nurses, and nurse practitioners) and Group 2—rehabilitation team (physical therapists, occupational therapists, and speech-language pathologists) who worked in the hospital setting and referred patients to outpatient and community rehabilitation services. 

### 3.1. Medical Team

#### 3.1.1. Demographics

A total of 19 HCPs from medical teams responded to the survey. Ten HCPs (52.6%) were nurses and nurse practitioners, and nine (47.3%) were oncologists and oncology residents. The majority of respondents were located in the provinces of Alberta (36.8%) and Ontario (31.5%) and worked in acute care or cancer hospitals (100%). The length of experience in the field ranged from 2 to 38 years (Table 1).

#### 3.1.2. Referrals to Rehabilitation Services (*N* = 19)

The average number of children and adolescents with cancer seen per year by the majority of the medical team respondents (74%) ranged from 21 to 100. Fifty-three percent of respondents indicated that they ‘often’ refer children to rehabilitation services; however, they also indicated a small number of children and adolescents referred to rehabilitation, ranging from 1–40, with 11–20 the most common response (36.8%).

Most medical team respondents reported referring children to rehabilitation services offered in an acute care hospital (52%), and 74% indicated that the majority of children and adolescents referred to rehabilitation received the service upon referral. Respondents indicated that a primary reason that some children did not end up receiving rehabilitation was related to parent/patient choice (Table 2).

#### 3.1.3. Reasons That Prompt Referral to Rehabilitation Services (*N* = 19)

Medical team respondents indicated that ‘surgery and/or amputation’ (*f* = 13/72), ‘peripheral neuropathy’ (*f* = 12/72), and ‘altered mobility’ (*f* = 11/72) were the top three reasons that prompted referral to rehabilitation services (Table 3).

#### 3.1.4. Availability of Rehabilitation Programs and Clinical Practice Guidelines (*n* = 19)

Seventy-nine percent of the medical team respondents reported having a rehabilitation program in their work setting. For those who reported not having a program, a lack of funding (33%) and limited availability of resources (17%) were listed as the most common reasons. Most respondents indicated that they do not follow any general pediatric oncology rehabilitation clinical practice guidelines (84%); however, the majority considered having recommendations to guide care very important and would very likely adopt guidelines or support their implementation in the future (63%) (Table 4).

#### 3.1.5. Barriers to Oncology Rehabilitation Programs (*N* = 19)

**Figure 2:**
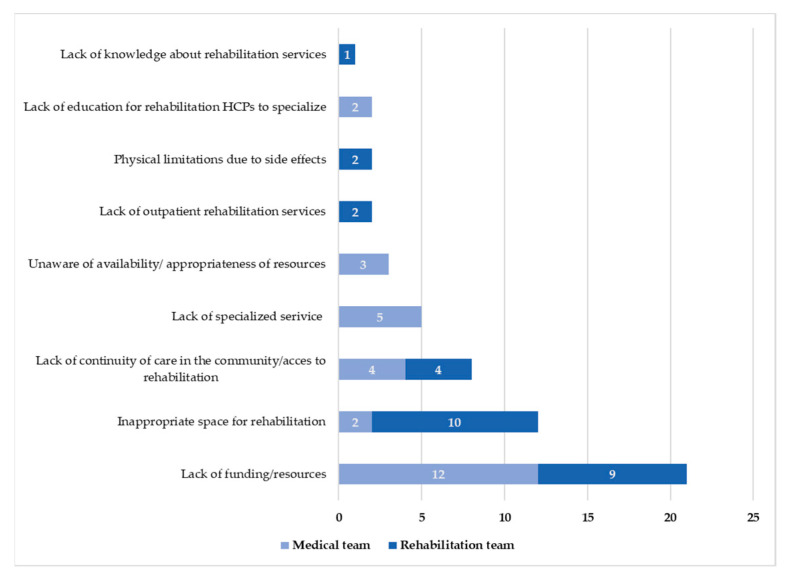
Frequency of reported barriers to rehabilitation programs reported by the medical and rehabilitation teams.

Respondents reported a large number of barriers (*f* = 29) to offering rehabilitation services. The most common barriers identified by the medical team included a lack of funding and resources (41%) and specialized oncology rehabilitation services (17%) (Figure 2).

### 3.2. Rehabilitation Team

#### 3.2.1. Demographics

A total of 15 HCPs from rehabilitation teams responded to the survey. Ten HCPs (67%) were physical therapists, four (27%) were occupational therapists, and one (7%) was a speech-language pathologist. The majority of respondents were located in the provinces of Alberta (40%), Quebec (26.7%), and Ontario (20%), and all worked in an acute care, cancer, or rehabilitation hospital (100%). The length of experience in the field ranged between 0.8 to 18 years (Table 1).

#### 3.2.2. Referrals to Rehabilitation Services (*N* = 15)

The average number of children and adolescents with cancer seen per year by the majority of the rehabilitation team respondents (73%) ranged between 1 and 49. Fifty three percent of respondents indicated that they ‘sometimes’ refer children to additional or alternate rehabilitation services—which is consistent with the reported referral to rehabilitation services, ranging from 1 to 40 children referred, with 6 to 10 the most common response (40%).

Most rehabilitation team respondents reported referring children to additional or alternate rehabilitation services in community/primary care settings (38%) and rehabilitation hospitals (35%), with 53% indicating that 75–100% of children and adolescents referred to additional or alternate rehabilitation services ended up receiving the service upon referral. Respondents also indicated that the reason why some children did not end up receiving additional or alternate rehabilitation was related to parent/patient choice (Table 2).

#### 3.2.3. Reasons That Prompt Referral to Rehabilitation Services (*N* = 15)

Rehabilitation team respondents indicated that ‘deconditioning’ (*f* = 6/25), ‘peripheral neuropathy’ (*f* = 4/25), and ‘weakness’ (*f* = 3/25) were the top three reasons that prompted referral to additional or alternate rehabilitation services (Table 3).

#### 3.2.4. Availability of Rehabilitation Programs and Clinical Practice Guidelines (*N* = 14)

Fifty percent of the rehabilitation team respondents reported having a rehabilitation program in their work setting, while those who did not have a program reported a lack of funding (30%) and availability of resources (40%) as the most common reasons. Half of respondents indicated that they do not follow any general pediatric oncology rehabilitation clinical practice guidelines (50%); however, the majority considered it very important to implement guidelines (71%) and, if available, would very likely adopt/support their implementation in the future (78%) (Table 4). 

#### 3.2.5. Barriers to Oncology Rehabilitation Programs (*N* = 14)

A large number of barriers were reported, with inappropriate space for rehabilitation (32%) and a lack of funding/resources (29%) as the most common barriers identified by the team (Figure 2; Table 5).

### 3.3. Thematic Findings to Inform a Framework for Action in Pediatric Oncology Rehabilitation

For the purposes of exploratory analyses, we identified three key perceived thematic barriers related to: (1) lack of dedicated funding and resources; (2) better access; and (3) the need for specialized pediatric oncology rehabilitation services (Table 5).

1. Dedicated funding and resources: Similar to the findings from the quantitative survey, both medical and rehabilitation professionals identified a lack of funding and resources related to staffing levels, space, and equipment. Specifically, medical team respondents identified the need for rehabilitation services in the outpatient and community settings. Additional comments on barriers from rehabilitation team respondents suggested that their work settings lack funding for appropriate equipment and availability of appropriate space to carry out rehabilitation interventions for children with cancer. Their comments suggested that (1) HCPs are working with the minimal resources available (e.g., no physical therapist available, adult-designed gymnasium, inadequate time for interventions), and that (2) children with cancer would benefit from age-appropriate spaces and equipment, as well as dedicated time for rehabilitation interventions.

2. Better access: During active cancer treatment, children may experience physical impairments, which can persist following completion of cancer treatment [34]. HCPs identified the need for routine screenings for common cancer-related impairments, from the time of diagnosis, to promote the timely identification of impairments amenable to rehabilitation services. Additional comments suggested that their work settings did not have oncology-specific rehabilitation services or outpatient rehabilitation services to provide continuity of care. Upon completion of cancer treatment, ensuring transitions of care from the inpatient rehabilitation team to outpatient or community health care teams was also seen as critical for optimal management of cancer-related impairments [34].

3. Specialized pediatric oncology services: Medical team respondents identified a lack of sufficiently trained rehabilitation staff to meet the needs of children with cancer. While it was acknowledged that rehabilitation professionals have skills and knowledge in the assessment and management of impairments related to musculoskeletal, neuromuscular, and cardiorespiratory systems, medical team respondents identified the need for rehabilitation HCPs with knowledge on the effects of cancer and its treatment on children and expertise in management of common effects in the context of cancer, such as chemotherapy-induced peripheral neuropathy. In contrast, rehabilitation team members identified a lack of understanding among doctors and nurses on the benefits of rehabilitation for children with cancer.

## 4. Discussion

To our knowledge, this is the first survey across Canada that explored rehabilitation service provisions and referral patterns for children and adolescents with cancer. While the majority of children with cancer may benefit from a rehabilitation program, our results suggest that children with cancer are primarily referred to rehabilitation services after major surgery where there is the potential for development of serious functional disabilities. Rehabilitation services may be beneficial for children with cancer across the cancer trajectory as a means to minimize the severity and long-term impact of treatment-related side effects [8]. According to Alfano et al. [35], oncology medical and nursing HCPs have limited knowledge about when and how to refer patients to rehabilitation services, resulting in low referral rates to rehabilitation services and consequently, a limited availability of oncology rehabilitation programs. Our findings are consistent with those of previously reported studies [25,26,27,31]. For example, Gohar et al. [31], conducted a retrospective chart review and a prospective questionnaire of frequency and rationale behind physicians’ referral of children with acute lymphoblastic leukemia to PT services at a children’s hospital in the United States. The authors reported that while physicians identified adverse musculoskeletal effects on chart review, only a third of children were referred to PT services [31]. Findings of the survey suggest the need for a multi-pronged approach to improving referrals including better physician awareness, training of rehabilitation HCPs in pediatric oncology, and the development of guidelines for referral to rehabilitation services. 

HCPs indicated that the main reason children and adolescents with cancer did not receive rehabilitation services was associated with parent/patient choice—similar to findings by Gohar et al. [31], who also reported ‘parent’s choice’, as well as ‘financial resources’ as reasons why children with cancer did not receive necessary rehabilitation. This finding may be attributed, in part, to the time burden experienced by parents in attending medical appointments and cancer treatments [36]; as such, rehabilitation may be seen as less of a priority than medical care. Previous studies have also identified poor compliance and attendance as the main challenges when providing rehabilitation for adolescents and young adults (AYAs) with cancer [37]. Other reasons parents and/or patients choose not to access rehabilitation may be (1) a lack of understanding on the part of parents on the benefits of rehabilitation programs in cancer and/or (2) limitations related to access of rehabilitation services close to home. Addressing the needs and wellbeing of families of children with cancer may facilitate uptake of rehabilitation programs. Prior research engaging parents in exercise and diet interventions has shown benefits for parents as well as positive outcomes in promoting long-term lifestyle changes in children [38]. Further research is needed to explore the needs and perspectives of parents and families towards pediatric oncology rehabilitation programs, as well as the barriers and facilitators to accessing services.

The majority of rehabilitation team respondents (38%) reported referring children and adolescents with cancer to community/primary care rehabilitation services that may or may not offer specialized services. A survey conducted in Australia compared confidence levels of physical therapists (*n* = 104) and exercise professionals (*n* = 32) treating adults versus AYAs with cancer and explored clinicians’ interest in specialized oncology education. Results showed that only 36% of physical therapists (*n* = 104) and exercise professionals (*n* = 32) reported confidence treating AYAs undergoing cancer treatment, and 57% reported feeling confident working with AYAs in remission post-treatment [37]. In contrast, 67% reported confidence treating adults undergoing cancer treatment, and 87% felt confident working with adults in remission post-treatment. Furthermore, only 5% of survey respondents reported they had received some form of education specific to AYA oncology, with 64% indicating interest in obtaining further education specific to AYA oncology. Thus, further exploration of the educational needs of HCPs delivering services to children with cancer, especially those working in community sites, may be warranted.

Similar to the results from a Canadian survey exploring adult oncology rehabilitation services [30], rehabilitation team respondents identified inappropriate space for rehabilitation and a lack of funding/resources as the main barriers to service provision. Additionally, in settings where no rehabilitation programs were available, HCPs reported limited availability of resources/space and funding as the reasons for the lack of rehabilitation services. Respondents also identified barriers related to the health system, namely the unrecognized need for, and support to develop and implement, pediatric oncology-specific clinical practice guidelines in rehabilitation. 

### 4.1. Future Directions 

**Figure 3:**
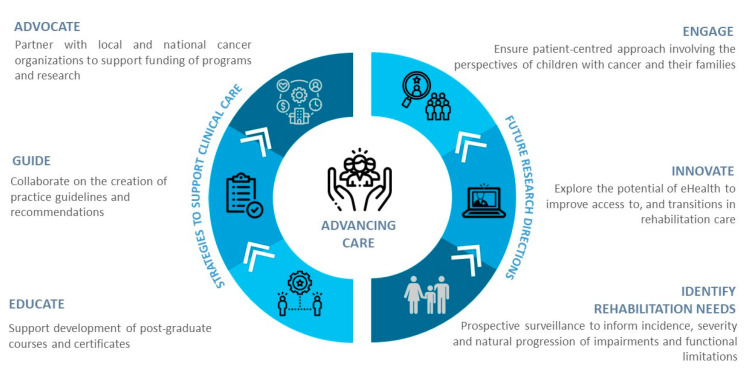
Framework to advance clinical care and guide future research in pediatric oncology rehabilitation.

We propose a framework comprising six key actionable strategies to advance clinical care and guide future research in pediatric oncology rehabilitation (Figure 3) [39].

1. *Advocate:* Families of children with cancer play a crucial role in advocating for improvements in cancer treatments, availability of resources, and advancements in research [40]. The creation of strong partnerships between patient advocacy groups as well as local and national non-profit cancer organizations are vital in accessing funds to advance rehabilitation care for children across the cancer trajectory [40].
2. *Guide:* The lack of high-quality rehabilitation-related research evidence is a current barrier to the development of clinical practice guidelines in pediatric oncology. A proposed interim step is for HCPs, researchers, and leaders in the field to collaborate on the creation of expert opinion guidelines and recommendations specific to pediatric oncology rehabilitation services.
3. *Educate:* Existing rehabilitation professional programs lack adequate content in oncology, and pediatric oncology, in particular [39]. To address this current gap, including pediatric oncology in clinical education programs and courses supporting oncology rehabilitation specialization and post-graduate certificates in cancer rehabilitation may serve to accelerate educational efforts in the field [41]. Education and resources are also needed for oncology medical teams to raise awareness of the benefits of rehabilitation.
4. *Identify:* Strategies such as prospective surveillance may help to inform the incidence, severity, and natural progression of impairments and functional limitations of the common childhood cancers [42]. As suggested by Alfano et al. [39], simply implementing a ‘brief patient questionnaire’ provided at oncology visits may allow HCPs to identify patients’ symptoms and make appropriate referrals to rehabilitation services.
5. *Innovate:* To address issues related to access to and transitions in care, research is needed evaluating the potential of eHealth. Telehealth and telecommunication technologies may offer opportunities for virtual delivery of rehabilitation interventions and surveillance for families living in rural and remote locations [41,43] or for those unable to travel to the health centers as a result of barriers such as time and illness [41].
6. *Engage:* To ensure a patient-centred approach, research is needed exploring the perspectives of parents and families towards pediatric oncology rehabilitation programs and family-reported barriers to and facilitators for accessing rehabilitation services.

### 4.2. Limitations

Several limitations were identified in this study. To protect the right of privacy and confidentiality, we did not ask personal information that identified participants. Therefore, we were not able to follow up with respondents when survey responses were unclear or incomplete. As well, although administered across the country, the survey did not encompass respondents from all provinces, which suggests that our results may not be representative of all pediatric oncology rehabilitation programs in Canada. Although a strength of the study involved the higher than anticipated response rate that exceeded our estimated sample size, the relatively small sample size is unlikely to be representative of all pediatric oncology HCPs across Canada. Our results, however, are similar to previous surveys of HCPs conducted in adults with cancer and those conducted in other jurisdictions [30,31].

## 5. Conclusions

Our study provides an overview of the current referral patterns and barriers to pediatric oncology rehabilitation services across Canada and proposes a framework to action to advance pediatric oncology rehabilitation and guide future research in a Canadian context. Despite limited research in the field, educational efforts specific to pediatric oncology rehabilitation are needed to build capacity for services across acute care and community/primary care locations. Prior to evaluating referral strategies to pediatric oncology rehabilitation programs, further research is needed to understand the needs and perspectives of children with cancer and their families toward rehabilitation services. Given the current paucity of research in pediatric oncology rehabilitation, and the barriers to care identified through this survey, action is needed to advance clinical practice and research efforts. 

## Figures and Tables

**Table 1:** Demographics.

	Overall *n* = 34, 100%	
	Medical Team *n* = 19, 55.8%	Rehabilitation Team *n* = 15, 44.1%
Province *(n, %)*		
Alberta	7 (36.8%)	6 (40%)
Ontario	6 (31.5%)	3 (20%)
British Columbia	4 (21%)	1 (6.7%)
Quebec	-	4 (26.7%)
Nova Scotia	2 (10.5%)	-
Not provided		1 (6.7%)
Professional designation *(n, %)*		
Physical therapists		10 (66.7%)
Nurse and nurse practitioners	10 (52.6%)	-
Oncologists and oncology residents	9 (47.3%)	-
Occupational therapists		4 (26.7%)
Speech-language pathologists		1 (6.7%)
Work setting *(n, %)*		
Acute care hospital	17 (89.5%)	10 (66.7%)
Cancer hospital	2 (10.5%)	3 (20%)
Rehabilitation hospital	-	2 (13.3%)
Years of experience *(n, %)*		
0.1–5	2 (10.5%)	3 (20%)
5.1–10	-	4 (26.7%)
10.1–15	4 (21%)	7 (46.7%)
15.1–20	5 (26.3%)	1 (6.7%)
20.1–30	6 (31.5%)	-
30.1–40	2 (10.5%)	-

**Table 2:** Referral patterns.

	Overall *n* = 34, 100%	
	Medical Team *n* = 19, 55.8%	Rehabilitation Team *n* = 15, 44.1%
Number of C&A seen per year (*n, %)*		
1–10		4 (26.7%)
11–20	1 (5.3%)	3 (20%)
21–49	6 (31.6%)	4 (26.7%)
50–100	8 (42.2%)	2 (13.3%)
>100	4 (21.1%)	2 (13.3%)
How often refer C&A to rehabilitation *(n, %)*		
Often	10 (52.6%)	4 (26.7%)
Sometimes	5 (26.3%)	8 (53.3%)
Rarely	4 (21.1%)	2 (13.3%)
Never		
Do not know		1 (6.7%)
Number of C&A referred to rehabilitation (*n, %)*		
1–5	4 (21.1%)	4 (26.7%)
6–10	4 (21.1%)	6 (40%)
11–20	7 (36.8%)	4 (26.7%)
21–40	4 (21.1%)	1 (6.7%)
Percentage of C&A that received rehabilitation (*n, %)*		
75–100%	14 (73.7%)	8 (53.3%)
50–75%	3 (15.8%)	3 (20%)
25–50%		1 (6.7%)
<25%	1 (5.3%)	
Don’t know	1 (5.3%)	3 (20%)
Location/type of service referred *(f, %)*		
Acute care hospital	16 (51.6%)	2 (6.9%)
Rehabilitation hospital	7 (22.6%)	10 (34.5%)
Community/primary care	6 (19.4%)	11 (37.9%)
Cancer hospital	2 (6.5%)	
Private practice		4 (13.8%)
Other		2 (6.9%)
Reasons why C&A referred did not receive rehabilitation *(n, %)*		
Parent/patient choice	5 (26.3%)	5 (33.3%)
Don’t know	4 (21.1%)	5 (33.3%)
Physical therapy was not deemed necessary	3 (15.8%)	1 (6.7%)
Financial resources	2 (10.5%)	2 (13.3%)
N/A–100% received	1 (5.3%)	1 (6.7%)
Other	4 (21.1%)	1 (6.7%)

Abbreviations: C&A, children and adolescents.

**Table 3:** Reasons that prompt referral to rehabilitation services.

	Overall *n= 34, 100%*	
	Medical Team *n = 19, 55.9%*	Rehabilitation Team *n = 15, 44.1%*
Surgery and/or amputation	13	2
Peripheral neuropathy	12	4
Altered mobility	11	2
De-conditioning	8	6
Weakness	7	3
Spinal cord injury	3	2
Bone tumour	3	-
Brain tumour	3	-
CNS tumour	3	-
Abnormal gross and fine motor skills	1	2
Neurological deficits	1	2
Bone marrow transplant	1	-
Tumour	1	-
Graft versus host disease of the joints	1	-
Extended hospital stays	1	-
Pain	1	-
Respiratory issues	1	-
Leukemia	1	-
Impaired balance	-	2
Total frequencies (*f)*	72	25

Abbreviations: CNS, central nervous system.

**Table 4:** Availability of rehabilitation programs and clinical practice guidelines.

	Overall	
	*n =* 33, 100%	
	Medical Team	Rehabilitation Team
	*n =* 19, 57.6%	*n =* 14, 42.4%
Work settings with a rehabilitation program *(n, %)*		
Yes	15 (78.9%)	7 (50%)
No	3 (15.8%)	6 (42.9%)
Do not know	1 (5.3%)	1 (7.1%)
Reasons for not having a rehabilitation program *(f, %)*		
Inadequate funding	2 (33.3%)	3 (30%)
Lack of availability of resources/space	1 (16.7%)	4 (40%)
Lack of rehabilitation professionals with experience in pediatric oncology	1 (16.7%)	-
Other–few admissions of children with cancer	1 (16.7%)	1 (10%)
Patients referred to rehabilitation programs that are not oncology specific	1 (16.7%)	2 (20%)
Lack of evidence to support rehabilitation interventions	-	-
Small pediatric oncology population	-	-
HCPs who follow pediatric oncology rehabilitation clinical practice guidelines *(n, %)*		
No	16 (84.2%)	7 (50%)
Yes	3 (15.8%)	5 (35.7%)
Do not know	-	2 (14.3%)

Abbreviations: HCP, healthcare professional.

**Table 5:** Thematic findings and representative quotes.

Themes	Medical Professional Quotes	Rehabilitation Professional Quotes
**Dedicated funding and resources**	*“Enthusiastic therapists who participate in team-based care. Not sure if we have appropriate facilities.”* *“(There are) Not enough resources. (Existing) Shared space that limits neutropenic patients from accessing gym if other patients (are) present.”* *“Off-therapy and after care clinics have no resources for physical rehabilitation.”* *“We are not funded for outpatient physical rehab(ilitation) services.”* *“OT (occupational therapy)/PT (physical therapy) have limited time with program (less than 50% of FTE (Full Time Equivalent) for PT (physical therapy) and less than that for OT (occupational therapy)).*	*“My clinic does not have enough resources and space to provide optimal rehabilitation programs for children and adolescents with cancer.”* *“Working in the public system funding is always a barrier.”* *“Not enough space in the clinic for rehabilitation making it difficult to provide service at times.”* *“Limited equipment and safety devices to challenge ped(iatric)s patients in a safe manner.”* *“We have no funding for the outpatient clinic, when we do try and cover there, we do not have adequate space at all.”* *“Human resources vs. needs and number of patients. Some patients under referred.”*
**Improved access and transitions in care**	*“Many of our children are from outside the region and do not have the services locally or ability to travel to the city for services. Our rehab (rehabilitation) hospital will not see patients while (children with cancer are) still on active treatment i.e., chemotherapy.”* * “Patients are from a wide geographical area. Access to PT (physical therapy)/OT (occupational therapy) is limited by where (the) patient lives, and resources that may be available in their home communities. Cost of travel (in time and money) can be a barrier for some families accessing rehab(ilitation) services.”* * “Patients on active therapy are not allowed to have services* via *our rehabilitation hospital.”* * “Outpatient access is more limited (same patients that were inpatients). Intensive programming through rehab facility is not usually accessible because of conflicts with ongoing cancer therapy.”*	*“At times when children still benefit from (an) active intervention, they may receive only consultation or may not be followed long term if they don’t have specific goals at that time.* *“A limitation is that we don’t always hear how the transfer to (the) community went or do not have a specific physiotherapist to speak with at the time of discharge.”* *“Referrals to community resources are specific to geographic region and there is a very high level of variation in the services provided.”* *“For children working on high level skills, the outpatient services tend to be less.”*
**Specialized** **rehabilitation** **services**	*“The physiotherapy team moves it’s PT’s (physical therapists) every few months to different units, so it is difficult for them to specialize.”* *“We do not have a specific program for this, we have one physiotherapist that has taken this patient group and did a lot of self-learning and advocacy to ensure the unique needs of this population are met. The hospital does not have a system that supports specialization for the physiotherapy group.”* *“We have (an) inpatient physiotherapist as part of our team. The outpatient setting is more difficult and has no dedicated physiotherapist.”*	*“Lack of knowledge of doctors / nurses on the contribution (of rehabilitation) especially in occupational therapy.”*

## Data Availability

The data on repository will be available on request to the corresponding author.

## Supplementary Materials

The following supporting information can be downloaded at: https://www.mdpi.com/article/10.3390/cancers15030693/s1, Table S1. Survey of Physical Rehabilitation in Children and Adolescents with Cancer.
