# Supplementary material for: Advancing Pediatric Oncology Rehabilitation: Survey Findings of Health Professionals’ Perceptions of Barriers to Care and a Framework for Action
Source: Cancers (Basel). 2023 Jan 23;15(3):693. doi: 10.3390/cancers15030693 (PMC9913711; doi:10.3390/cancers15030693)
Supplement: Supplementary file 1 [file cancers-15-00693-s001.zip › cancers-2168586-supplementary.pdf]

Physical rehabilitation in children and adolescents with cancer

This survey will take approximately 15-20 minutes to complete. You are allowed to stop the survey at any time and return to it later. In order to save your answers and to continue where you left off, you must scroll down to the end of the page and click the "Save and Return Later" button.

If you do not complete the questionnaire, a code will be automatically created to you. Please write it down. You may bookmark this page to return to the survey, OR you can have the survey link emailed to you by providing your email address. When you are ready to return, please click on the link provided to you. You will be asked to type the code to access your questionnaire.

For any questions or concerns, please contact us:

Paula Ospina, MSc Student: pospina@ualberta.caDr. Margaret L. McNeely: mmcneely@ualberta.caDr. David Eisenstat: eisensta@ualberta.caDr. Lesley Wiart: lwiart@ualberta.ca

The aim of this survey is to learn more about the current physical rehabilitation practices and service provision offered by healthcare professionals working with children and adolescents with cancer (0 to 19 years-old) across Canada.

Please remember that the information collected in this survey will be used solely for academic purposes. Any information you disclose on this survey will be anonymous. If the results of this study are published, your identity will remain confidential.

SECTION 1. DEMOGRAPHIC INFORMATION

In what city do you work?

____

In what province/territory do you work?

____

What is your professional designation?

- ◯ Oncologist
- ◯ Nurse
- ◯ Physiotherapist
- ◯ Occupational therapist
- ◯ Other

(Please select one that applies, if your answer is not stated, select "Other" and provide your designation)

Other:

____

(Please indicate your professional designation)

In which setting do you work?

- ◯ Acute Care Hospital
- ◯ Cancer hospital
- ◯ Private practice
- ◯ Community/Primary Care
- ◯ Rehabilitation hospital
- ◯ Other

(Please select one that applies, if your answer is not stated, select "Other" and provide your type of work setting)

Other:

____
(Please indicate your type of work setting)

How long have you been working with children and adolescents with a diagnosis of cancer?

- ◯ Months
- ◯ Years

(Please indicate in which unit you would like to provide your answer)

Please type the number of MONTHS:

____

Please type the number of YEARS:

____

Please indicate if you PROVIDE or REFER children and adolescents with cancer to PHYSICAL REHABILITATION?

- ◯ Provide
- ◯ Refer
- ◯ Both

How many children and adolescents with cancer do you see on average per year?

- ◯ 1 - 5
- ◯ 6 - 10
- ◯ 11 - 20
- ◯ > 20 (if greater than 20 please provide the number)

Total number:

____

HOW OFTEN do you refer children and adolescents with cancer TO PHYSICAL REHABILITATION?

- ◯ Often
- ◯ Sometimes
- ◯ Rarely
- ◯ Never
- ◯ Don't know

On average, how many children and adolescents with cancer do you REFER to PHYSICAL REHABILITATION SERVICES per YEAR?

- ◯ 1 - 5
- ◯ 6 -10
- ◯ 11 - 20
- ◯ > 20 (if greater than 20 please provide the number)

Total number:

____

To which location/ type of service do you REFER children and adolescents with cancer?

- ☐ Community/Primary Care
- ☐ Rehabilitation hospital
- ☐ Cancer hospital
- ☐ Private practice
- ☐ Acute Care Hospital
- ☐ Other

Other:

____

What PERCENTAGE of children and adolescents with cancer referred to PHYSICAL REHABILITATION, actually DID RECEIVE physical rehabilitation?

- ◯ 75-100%
- ◯ 50-75%
- ◯ 25-50%
- ◯ < 25%
- ◯ Don't know

What are the REASONS why children and/or adolescents referred DID NOT RECEIVE physical rehabilitation?	◯ Parents choice<br>◯ Financial resources<br>◯ Physiotherapist did not deem necessary<br>◯ Don't know<br>◯ Other<br>(Please select one that applies, if your answer is not stated, select "Other" and provide the reason(s))
Other	__________________________________
Which CANCER SIDE EFFECTS in children and adolescents will prompt you to refer to PHYSICAL REHABILITATION SERVICES?	____

SECTION 2. PHYSICAL REHABILITATION INTERVENTIONS

On average, how many children and adolescents with cancer do you provide PHYSICAL REHABILITATION to per YEAR?

- ◯ 1 - 5
- ◯ 6 -10
- ◯ 11 - 20
- ◯ > 20 (if greater than 20 please provide the number)

Total number: ______________________________

SIDE EFFECTS related to cancer and its treatments

Which of the following side effects do you consider should be the HIGHEST PRIORITY for physical rehabilitation in children and adolescents with cancer?

If your answer is not stated, select "Other" and provide your answer

- ◯ Cancer-related fatigue
- ◯ Gait alterations
- ◯ Pain
- ◯ Muscle weakness
- ◯ Decrease in flexibility
- ◯ Alteration in motor performance
- ◯ Limitation in Activities of Daily Living
- ◯ Balance alterations
- ◯ Proprioception alterations
- ◯ Peripheral neuropathy
- ◯ Motor development alterations
- ◯ Muscular stiffness
- ◯ Other

Other: __________________________________

Which of the following side effects do you consider should be the SECOND PRIORITY for physical rehabilitation in children and adolescents with cancer?

If your answer is not stated, select "Other" and provide your answer

- ◯ Cancer-related fatigue
- ◯ Gait alterations
- ◯ Pain
- ◯ Muscle weakness
- ◯ Decrease in flexibility
- ◯ Alteration in motor performance
- ◯ Limitation in Activities of Daily Living
- ◯ Balance alterations
- ◯ Proprioception alterations
- ◯ Peripheral neuropathy
- ◯ Motor development alterations
- ◯ Muscular stiffness
- ◯ Other

Other: __________________________________

Which of the following side effects do you consider should be the THIRD PRIORITY for physical rehabilitation in children and adolescents with cancer?

If your answer is not stated, select "Other" and provide your answer

- ◯ Cancer-related fatigue
- ◯ Gait alterations
- ◯ Pain
- ◯ Muscle weakness
- ◯ Decrease in flexibility
- ◯ Alteration in motor performance
- ◯ Limitation in Activities of Daily Living
- ◯ Balance alterations
- ◯ Proprioception alterations
- ◯ Peripheral neuropathy
- ◯ Motor development alterations
- ◯ Muscular stiffness
- ◯ Other

Other: __________________________________

In which setting do you provide PHYSICAL REHABILITATION to children and adolescents with cancer?

- ◯ Inpatient
- ◯ Outpatient
- ◯ Both

INPATIENT physical rehabilitation interventions

What types of PHYSICAL REHABILITATION are you providing for children and adolescents with cancer?

Select all that apply, if your answer is not stated, select "Others" and provide the type(s) of intervention(s).

	BEFORE CANCER TREATMENT	DURING CANCER TREATMENT	AFTER CANCER TREATMENT
Passive/Active/Active assistive range of motion (ROM) exercises	☐	☐	☐
Joint mobilizations and manipulations	☐	☐	☐
Manual therapy/distraction techniques	☐	☐	☐
Myofascial release	☐	☐	☐
Soft tissue massage	☐	☐	☐
Trigger point therapy	☐	☐	☐
Deep transverse friction	☐	☐	☐
Flexibility exercises	☐	☐	☐
Balance training	☐	☐	☐
Proprioception exercises	☐	☐	☐
Theraband/theratubing strengthening exercises	☐	☐	☐
Free weights strengthening exercises	☐	☐	☐
Functional strengthening	☐	☐	☐
Aerobic exercise using treadmill	☐	☐	☐
Aerobic exercise using cycle ergometer	☐	☐	☐
Aerobic exercise using arm ergometer	☐	☐	☐
Gait training/re-education	☐	☐	☐
Aquatic therapy	☐	☐	☐
Hydrotherapy	☐	☐	☐
Bandaging techniques	☐	☐	☐
Proprioceptive Neuromuscular Facilitation (PNF)	☐	☐	☐
Kinesio taping	☐	☐	☐
Education	☐	☐	☐
Other	☐	☐	☐

Please indicate other type(s) of physical intervention(s) you provide BEFORE CANCER TREATMENT: ________________________________

Please indicate other type(s) of physical intervention(s) you provide DURING CANCER TREATMENT: ________________________________

Please indicate other type(s) of physical intervention(s) you provide AFTER CANCER TREATMENT ________________________________

Do you apply any of the following PHYSICAL AGENTS to treat the side effects of cancer and its treatment(s) in children with any type of cancer?<br><br>Select all that apply, if your answer is not stated, select "Others" and provide the type(S) of PHYSICAL AGENT(S).	☐ None<br>☐ Electrical stimulation<br>☐ Ultrasound<br>☐ Cryotherapy (e.g. cold packs)<br>☐ Thermotherapy (e.g. hot packs)<br>☐ Infrared light therapy<br>☐ Ultraviolet therapy<br>☐ Diathermy<br>☐ Paraffin wax bath<br>☐ Transcutaneous Electrical Nerve Stimulation (TENS)<br>☐ Other
Other:	__________________________________<br>(Please indicate other type(s) of PHYSICAL AGENT(S) you provide)
IF you selected any of the previous physical agents, please provide a brief RATIONALE FOR USE in children and adolescents with cancer.	__________________________________

OUTPATIENT physical rehabilitation interventions

What types of PHYSICAL REHABILITATION are you providing to treat or help children presenting with cancer?

Select all that apply, if your answer is not stated, select "Others" and provide the type(s) of intervention(s).

	BEFORE CANCER TREATMENT	DURING CANCER TREATMENT	AFTER CANCER TREATMENT
Passive/Active/Active assistive range of motion (ROM) exercises	☐	☐	☐
Joint mobilizations and manipulations	☐	☐	☐
Manual therapy/distraction techniques	☐	☐	☐
Myofascial release	☐	☐	☐
Soft tissue massage	☐	☐	☐
Trigger point therapy	☐	☐	☐
Deep transverse friction	☐	☐	☐
Flexibility exercises	☐	☐	☐
Balance training	☐	☐	☐
Proprioception exercises	☐	☐	☐
Theraband/theratubing strengthening exercises	☐	☐	☐
Free weights strengthening exercises	☐	☐	☐
Functional strengthening	☐	☐	☐
Aerobic exercise using treadmill	☐	☐	☐

Aerobic exercise using cycle ergometer	☐	☐	☐
Aerobic exercise using arm ergometer	☐	☐	☐
Gait training/re-education	☐	☐	☐
Aquatic therapy	☐	☐	☐
Hydrotherapy	☐	☐	☐
Bandaging techniques	☐	☐	☐
Proprioceptive Neuromuscular Facilitation (PNF)	☐	☐	☐
Kinesio taping	☐	☐	☐
Education	☐	☐	☐
Other	☐	☐	☐

Please indicate other type(s) of physical intervention(s) you provide BEFORE CANCER TREATMENT: ______________________

Please indicate other type(s) of physical intervention(s) you provide DURING CANCER TREATMENT: ______________________

Please indicate other type(s) of physical intervention(s) you provide AFTER CANCER TREATMENT ______________________

Do you apply any of the following PHYSICAL AGENTS to treat the side effects of cancer and its treatment(s) in children with cancer?

Select all that apply, if your answer is not stated, select "Others" and provide the type(S) of PHYSICAL AGENT(S).

- ☐ None
- ☐ Electrical stimulation
- ☐ Ultrasound
- ☐ Cryotherapy (e.g. cold packs)
- ☐ Thermotherapy (e.g. hot packs)
- ☐ Infrared light therapy
- ☐ Ultraviolet therapy
- ☐ Diathermy
- ☐ Paraffin wax bath
- ☐ Transcutaneous Electrical Nerve Stimulation (TENS)
- ☐ Other

Other:

____

(Please indicate other type(s) of PHYSICAL AGENT(S) you provide)

IF you selected any of the previous physical agents, please provide a brief RATIONALE FOR USE in children and adolescents with any type of cancer. ______________________

Please check the statement that best describes your physical rehabilitation intervention:

- ○ All patients presenting SIMILAR symptoms receive a similar intervention
- ○ All patients presenting DIFFERENT symptoms receive a similar intervention
- ○ Each patient receives an individualized intervention according to their needs

Do you provide interventions to children and adolescents with cancer in GROUPS or INDIVIDUALLY?	◯ Groups<br>◯ Individually<br>◯ Both<br>◯ Other
Other:	__________________________________
Do you BELIEVE that the physical rehabilitation interventions you provide help to REDUCE the burden of CANCER SIDE EFFECTS?<br><br>Please select one that applies, if your answer is not stated, select "Other" and provide your answer	◯ Yes<br>◯ No<br>◯ Other
Other:	__________________________________
Is there anything about your physical rehabilitation program for children and adolescents with cancer that you would like to IMPROVE or ADD?	◯ Yes<br>◯ No
If you answered YES, please add the information:	____

OUTCOME MEASURES USED IN PHYSICAL REHABILITATION

Which TESTS or OUTCOMES MEASURES do you use to ASSESS and/or MONITOR the progress in children and adolescents with cancer?

AEROBIC CAPACITY

Please select all that apply, if your answer is not stated, select "Other" and provide your answer

- ☐ 6-min-Walk-test
- ☐ 9-min-Walk-test
- ☐ Timed Up and Down Stairs (TUDS)
- ☐ YMCA test
- ☐ Rockport test
- ☐ None
- ☐ Other

Other: ______________________________

GAIT

Please select all that apply, if your answer is not stated, select "Other" and provide your answer

- ☐ Electronic gait analysis
- ☐ Manual gait analysis (e.g. walking on a brown wrapping paper with feet covered in talcum powder)
- ☐ Dynamic Gait Index
- ☐ None
- ☐ Other

Other: ______________________________

POSTURE

Please select all that apply, if your answer is not stated, select "Other" and provide your answer

- ☐ Visual analysis
- ☐ None
- ☐ Other

Other: ______________________________

BALANCE

Please select all that apply, if your answer is not stated, select "Other" and provide your answer

- ☐ Berg Balance Scale (BBS)
- ☐ Romberg test
- ☐ Paediatric Balance Scale (PBS)
- ☐ The Flamingo Balance Test
- ☐ Tinetti balance assessment tool
- ☐ None
- ☐ Other

Other: ______________________________

STRENGTH

Please select all that apply, if your answer is not stated, select "Other" and provide your answer

- ☐ Manual muscle testing
- ☐ Hand-held MYOMETRY
- ☐ Hand-held DYNAMOMETRY
- ☐ Biodex
- ☐ The spring scale
- ☐ The lateral step-up test
- ☐ The sit-to-stand test
- ☐ The up-and-down stairs test
- ☐ The minimum chair height test
- ☐ The incremental shuttle walking test
- ☐ None
- ☐ Other

Other: ______________________________

FLEXIBILITY/JOINT RANGE OF MOTION<br><br>Please select all that apply, if your answer is not stated, select "Other" and provide your answer	☐ Goniometry<br>☐ Sit and reach test<br>☐ None<br>☐ Other
Other:	______________________________
MOTOR DEVELOPMENT<br><br>Please select all that apply, if your answer is not stated, select "Other" and provide your answer	☐ Test of Gross Motor Development (TGMD)<br>☐ Bruininks Osteretsky Test of Motor Proficiency (BOT)<br>☐ Movement Assessment Battery for Children (MABC)<br>☐ Peabody Developmental Motor Scales (PDMS-2)<br>☐ Alberta Infant Motor Scale (AIMS)<br>☐ Miller Function and Participation Scales (MFUN-PS)<br>☐ None<br>☐ Other
Other:	______________________________
FUNCTIONAL ABILITIES<br><br>Please select all that apply, if your answer is not stated, select "Other" and provide your answer	☐ Functional Mobility Assessment (FMA)<br>☐ Pediatric Evaluation of Disability Inventory (PEDI)<br>☐ Functional Independence Measure for Children (WeeFIM)<br>☐ Vineland Adaptive Behavior Scale<br>☐ Timed Up and Go (TUG)<br>☐ None<br>☐ Other
Other:	______________________________
QUALITY OF LIFE<br><br>Please select all that apply, if your answer is not stated, select "Other" and provide your answer	☐ Pediatric Quality of Life Inventory (PedsQL)<br>☐ Child Health Questionnaire (CHQ)<br>☐ DISABKIDS Chronic Generic Measure - 37 (DCGM)<br>☐ DISABKIDS Chronic Generic Measure - 12 (DCGM)<br>☐ KINDL-R<br>☐ None<br>☐ Other
Other:	______________________________
PAIN<br><br>Please select all that apply, if your answer is not stated, select "Other" and provide your answer	☐ Faces Pain Scale<br>☐ Visual Analog Scale (VAS)<br>☐ None<br>☐ Other
Other:	______________________________

SENSORY FUNCTION<br><br>Please select all that apply, if your answer is not stated, select "Other" and provide your answer	☐ Myofilament test<br>☐ Hot and cold test/temperature test<br>☐ Sharp and dull test<br>☐ Vibration test<br>☐ None<br>☐ Other
Other:	__________________________
FATIGUE<br><br>Please select all that apply, if your answer is not stated, select "Other" and provide your answer	☐ Rating of Perceived Exertion (RPE)<br>☐ Kids Fatigue Severity Scale (K-FSS)<br>☐ PedsQL Multidimensional Fatigue Scale<br>☐ Childhood Cancer Fatigue Scale (CCFS)<br>☐ Fatigue Scale for a child (FS-C)/ adolescents (FS-A)/ for parents (FS-P)<br>☐ None<br>☐ Other
Other:	__________________________
PERIPHERAL NEUROPATHY<br><br>Please select all that apply, if your answer is not stated, select "Other" and provide your answer	☐ Paediatric Modified Total Peripheral Neuropathy Score (ped-mTNS)<br>☐ Total Neuropathy Score-Pediatric Vincristine (TNS-PV)<br>☐ Total Neuropathy Score (TNS)<br>☐ None<br>☐ Other
Other:	__________________________

SECTION 3. AVAILABLE GUIDELINES AND CURRENT BARRIERS IN THE HEALTH SYSTEM

Does your work setting have a PHYSICAL REHABILITATION program in Paediatric Oncology?

- ◯ Yes
- ◯ No
- ◯ Don´t know

If YES, please provide a brief description about it:

____

If NO, please select the possible reasons:

- ☐ Small paediatric oncology population
- ☐ Lack of evidence to support physical rehabilitation interventions
- ☐ Lack of physical rehabilitation professionals with experience in paediatric oncology
- ☐ Availability of resources/space
- ☐ Funding
- ☐ Patients referred to rehabilitation programs that are not oncology specific
- ☐ Other

Other:

____

Do you follow any clinical practice guidelines in PHYSICAL REHABILITATION?

- ◯ Yes
- ◯ No

If YES, please provide a brief description about it:

____

Do you have any resources that you are willing to share?

Are there any FACILITATORS (ALREADY EXISTING) in your work setting that support offering services in PHYSICAL REHABILITATION for children and adolescents with cancer?

Please indicate any facilitators

(e.g. "My clinical setting has an appropriate facility and equipment for physical rehabilitation for children")

____

Are there any BARRIERS in your work setting that may impact your ability to offer PHYSICAL REHABILITATION PROGRAMS for children and adolescents with cancer?

Please indicate any barriers

(e.g. "My clinical setting does not have enough resources for rehabilitation for children with cancer")

____

How important do you consider it is to IMPLEMENT clinical practice guidelines in PHYSICAL REHABILITATION for children and adolescents with cancer?

- ◯ Very important
- ◯ Moderately important
- ◯ Slightly important
- ◯ Not at all important

In the FUTURE, how likely are you to ADOPT/SUPPORT the implementation of clinical practice guidelines in PHYSICAL REHABILITATION INTERVENTION for children and adolescents with cancer?	◯ Very likely<br>◯ I don't know<br>◯ Not likely
When the study is complete, would you like to receive a summary of the findings?	◯ Yes<br>◯ No
Email:	__________________________
Additional comments or suggestions of this survey	________________________________
